# InforMD: a new initiative to raise public awareness about breast density

**DOI:** 10.3332/ecancer.2018.807

**Published:** 2018-02-06

**Authors:** Honor J Hugo, Aneta Zysk, Pallave Dasari, Kara Britt, John L Hopper, Jennifer Stone, Erik W Thompson, Wendy V Ingman

**Affiliations:** 1 Institute of Health and Biomedical Innovation, Queensland University of Technology, Kelvin Grove 4059, Australia; 2 School of Biomedical Sciences, Faculty of Health, Queensland University of Technology, Gardens Point 4000, Australia; 3 Translational Research Institute, Woolloongabba 4102, Australia; 4 Discipline of Surgery, School of Medicine, The Queen Elizabeth Hospital, University of Adelaide, Woodville 5011, Australia; 5 The Robinson Research Institute, University of Adelaide, Adelaide 5000, Australia; 6 Peter MacCallum Cancer Centre, Melbourne 3000, Australia; 7 The Sir Peter MacCallum Department of Oncology, University of Melbourne, Melbourne VIC 3000, Australia; 8 Centre for Epidemiology and Biostatistics, Melbourne School of Population and Global Health, The University of Melbourne, Parkville 3052, Australia; 9 Centre for Genetic Origins of Health and Disease, Curtin University and the University of Western Australia, Perth 6000, Australia

**Keywords:** breast density, mammographic density, public awareness, television, social media, mammography, breast, radiologists

## Abstract

On a mammogram, breast density (also known as mammographic density) is shown as white and bright regions and is associated with reduced sensitivity in cancer detection and increased breast cancer risk. However, many Australian women are unaware of the significance of breast density as it is not routinely reported or discussed. In order to address this lack of knowledge, Australian breast cancer researchers with expertise in mammographic density formed the InforMD alliance (INformation FORum on Mammographic Density) in 2016. The alliance is working to raise awareness of breast density with the goal of improving breast cancer diagnosis and health outcomes for women. The InforMD website (www.InforMD.org.au) was launched in October 2016, coinciding with a major nationwide public awareness campaign by the alliance during breast cancer awareness month. The website contains unbiased, accurate, updated information on breast density. The website also provides summaries of major research articles in layperson language, recent news items related to breast density, links to relevant information for health professionals, events, and feature articles. Members of the public and health professionals can also subscribe for news updates. The interactive online Forum section facilitates discussion between health professionals, scientists and members of the public. To increase online traffic to the website, Facebook (www.facebook.com/BeInforMD) and Twitter (https://twitter.com/BeInforMD_) pages were launched in December 2016. Since its launch, InforMD has generated considerable interest. The public awareness campaign reached over 7 million Australians through a combination of newspaper, TV, radio, and online news. The website has attracted 13,058 unique visitors and 30,353 page views (data as of 19/12/2017). Breast cancer researchers have a significant role to play in disseminating information to the public on breast density. A combination of mainstream and social media, together with a well-informed and updated website, has laid the groundwork for the InforMD alliance to reach a wide audience.

## Introduction

On a mammogram, breast density (also known as mammographic density) is shown as white and bright regions. The American College of Radiology describes four categories of density in the BI-RADS Atlas (5th Edition) ranging from ‘Mostly fatty’ to ‘Extremely dense’ [1]. Almost 8% of women aged between 40 and 74 years have ‘Extremely dense’ breasts, and 35% have ‘Heterogeneously dense’ breasts [2]. The sensitivity of mammography to detect breast cancer is reduced in women with dense breasts, as potential tumours, which are shown as white on a mammogram, can be masked by the white dense regions [3]. It is also known that breast cancer is more likely to develop in women with dense breast tissue. Women with breasts classified as ‘Extremely dense’ are 4–6 times more likely to develop breast cancer compared to women of the same age and body mass index whose breasts are classified as ‘Mostly fatty’ [4].

The current position of BreastScreen Australia is not to notify women if they have dense breasts [5], and the Royal Australian and New Zealand College of Radiologists (RANZCR) recommend against reporting breast density to women [6]. The policy of non-reporting by BreastScreen and RANZCR is primarily linked to the lack of clear evidence that supplementary screening approaches, such as ultrasound or magnetic resonance imaging, ultimately provide a breast cancer survival benefit on a population basis [5, 6]. More breast cancers are discovered by these methods, but they have issues around cost and accessibility, and ultrasound carries a high false positive rate. There are also complexities around the accurate measurement of density by radiologists [7, 8].

The national BreastScreen Australia program is funded and run by individual states, and this can result in variations in policy between states. In one state in Australia, Western Australia, the BreastScreen program does notify women with high density that their screening mammogram has reduced sensitivity [9] and provides information to healthcare professionals to guide screening decisions [10]. However, due to the lack of reporting of breast density nationally, many Australian women are not aware of this factor and how it affects mammogram sensitivity or cancer risk.

The InforMD (INformation FORum on Mammographic Density) alliance was established by breast cancer researchers across Australia, in South Australia (Associate Professor Wendy Ingman), Western Australia (Dr Jennifer Stone), Victoria (Professor John Hopper and Dr Kara Britt), and Queensland (Professor Erik (Rik) Thompson and Dr Honor Hugo). The alliance was created following discussion at a national biennial scientific meeting ‘Why Study Mammographic Density?’ held in August 2016. At this time, there was little information on Australian websites informing women about breast density and a strong message from consumers present that they would like more information. It was collectively appreciated that the problem of breast density contributing to missed cancers by mammography was serious, such that it warranted the unprecedented action of research scientists to form a national alliance, step out of their labs, and ‘start a conversation’ with the Australian community.

The aim of InforMD is to discover, provide and discuss the most current information on breast density and keep Australians up to date on new information as it becomes available. Furthermore, InforMD aims to improve prevention and early detection of breast cancer through increased understanding and better utilisation of breast cancer screening in Australia to lower the impact of this disease.

## Methods and results

The InforMD website (www.InforMD.org.au) was launched on 4th of October 2016 to coincide with National Breast Cancer Awareness month. The website contains unbiased, accurate, updated information on breast density. The website also provides summaries of major research articles in layperson language, recent news items related to breast density, links to relevant information for health professionals, events and feature articles. Members of the public and health professionals can subscribe for news updates. The interactive online Forum section facilitates discussion between health professionals, scientists, and members of the public. To increase online traffic to the website, Facebook (www.facebook.com/BeInforMD) and Twitter (https://twitter.com/BeInforMD_) pages were launched in December 2016. The website has attracted 13,058 unique visitors and 30,353 page views (data as of 19/12/2017). Referring websites are primarily Facebook and Google, and the FAQs page is the most popular webpage, after the Homepage.

Launch of the InforMD website was accompanied by a national media campaign regarding the significance of breast density in detection of breast cancer. The alliance issued a media release through the University of Adelaide on 4th of October, with each member of InforMD available for interview throughout the day, and additional media releases made from each of our institutions. The media campaign generated considerable interest, and reached over 7 million Australians through a combination of radio, television, online and print news (Table 1). Notably, a media exclusive with News Corp associated press resulted in publication in nine newspapers with a combined circulation of 1.1 million, and coverage by television news stations resulted in 29 items reaching 4.8 million viewers.

The InforMD alliance also authored an article in *The Conversation* [11] published on 4th of October 2016. This article has reached 12,157 readers, was shared 328 times on social media, and republished on seven other websites including Mamamia.com and IFLScience.com (data as of 19/12/2017).

## Discussion

The InforMD alliance was formed on the cusp of a wave of public interest across the globe in breast density. This began with the advocacy work by Dr Nancy Cappello, a breast cancer survivor with dense breasts, who spearheaded the Breast Density Notification law in the State of Connecticut in 2009. To date, 30 other states have followed suit [12]. Dr Cappello continues her work through her website, irreverently named ‘Are you Dense?’, which is run by consumer advocates. The Facebook page associated with the *Are You Dense* website currently attracts over 5,000 followers. Many other countries are keenly observing the impact of the US density reporting mandate, also reflected in the number of websites and associated Facebook and Twitter pages and blogs run by consumer advocates who feel very strongly that women should know about this issue. These include websites from Canada (www.densebreastscanada.ca), Ireland (https//beingdense.com) and the UK (facebook: Breast Density Matters UK). Other US breast density sites are run by a medical advisory board comprising radiologists and other breast cancer specialists, working alongside consumer advocates. These sites www.Dense-info.org and www.breastdensity.info aim to provide accurate information on breast density and the associated breast cancer risk, and update the general public on new discoveries in this arena. The InforMD website is comparable to these websites in this resolve to provide balanced, evidence-based information.

The breast cancer researchers behind InforMD believe that progress towards reporting breast density involves a shared conversation between government, healthcare professionals, scientists and, most importantly, women. To this end, the InforMD alliance is currently advocating for establishment of a multi-disciplinary committee involving all stakeholders, with the mandate of development of guidelines for density reporting. These national Australian breast density reporting guidelines would provide evidence-based and consensus-based information needed to achieve best practise, whilst reflecting the community’s range of attitudes and concerns.

Introduction of Breast Density Notification Laws in 31 states of the USA has been initiated largely by patient advocates who have had a breast cancer missed on a screening mammogram and wished to prevent the same from happening to others. In some cases, these patient advocates have died of metastatic breast cancer which might possibly have been avoided if they had been notified of the reduced sensitivity of their mammogram due to high breast density. Changes to health policy driven by introduction of state legislation can result in public mistrust of governing medical bodies such as the US Preventative Services Task Force, significant alarm and anxiety in women [13], and a confusing transition phase where the medical fraternity is not sufficiently informed as to the best advice to provide their patients. This situation may have been mitigated somewhat if patients had been included early on in decisions by healthcare professionals around reporting breast density.

In the 12 months since the inception of InforMD, there has been considerable public conversation in Australia around breast density. InforMD has continued to provide unbiased and accurate information to government, healthcare professionals and women, to help inform this complex conversation. We attribute our success in stimulating this dialogue to the clear consistent message we promote, the inclusive respectful approach we have taken in our conversations with all stakeholders, and that the message comes from an alliance of breast cancer researchers rather than from an individual.

The InforMD alliance is a unique and unprecedented model for breast density awareness and advocacy. In Australia, this scientist-led advocacy group is raising awareness of this important and complex issue, and promoting conversation between doctors and patients about the best way to report breast density. From an international perspective, the InforMD alliance provides up-to-date, accurate and unbiased information about new breast density research on its website and through social media. We welcome involvement in website content from researchers, healthcare professionals and women. It is anticipated that the website will continue to grow as the conversation on breast density continues.

## Conclusions

As public interest builds on this issue, the InforMD alliance will continue to serve the general public by communicating new research in the field and promoting dialogue between government, healthcare professionals and women.

## Conflicts of interest

The authors have no conflicts of interest to declare.

## Authors’ contributions

HJH and WVI drafted the manuscript, and all other authors contributed to the final version. All authors contributed to the InforMD public awareness campaign and development of the InforMD website.

## Figures and Tables

**Table 1.** Summary of Australian public awareness campaign media reach.

Medium	No. of items	No. of people reached
Newspaper	9	1,100,000
TV news	29	4,800,000
Radio	9	1,100,000
Online news	10	300,000
TOTAL	57	7,300,000
